# Barriers and facilitators of Patient-Public Engagement for health system improvement in Sub-Saharan Africa: A systematic scoping review

**DOI:** 10.1016/j.hpopen.2021.100055

**Published:** 2021-10-07

**Authors:** Samuel Egyakwa Ankomah, Adam Fusheini, Sarah Derrett

**Affiliations:** a Department of Preventive and Social Medicine, University of Otago, Dunedin, New Zealand; b Centre for Health Literacy and Rural Health Promotion, Accra, Ghana

**Keywords:** Patient-public engagement, Barriers and facilitators, Health system improvement, Community engagement, Sub-Saharan Africa

## Abstract

**Introduction:**

Patient-Public Engagement (PPE) is central to most community and public health interventions. There are reports on PPE’s impact on improving health and health systems. Yet, PPE initiatives are infrequent in Sub-Saharan Africa (SSA). A key step to enhancing engagement is identifying facilitators and barriers of PPE. Evidence synthesis of PPE’s effect on improving health systems is lacking. This study seeks to address this knowledge gap.

**Methods:**

This review (Protocol published) followed Arksey and O’Malley’s guidelines for conducting and reporting scoping reviews. A systematic search of peer-reviewed English language literature published between January 1999 and December 2019 on Scopus, Medline (Ovid), CINAHL and Embase databases was conducted. A thematic framework synthesis was employed.

**Results:**

Eighteen articles from ten Sub-Saharan African countries met the inclusion criteria of studies focusing on patient, public, citizen or community consultation/engagement/involvement in health services in Sub-Saharan Africa; as well as on barriers and facilitators for health systems improvement. The identified barriers and facilitators for health systems improvement were categorised onto a framework comprising individual-level, community-level and macro/strategic-level factors. Previous reviews on PPE have not focused on barriers and facilitators and its effect on improving health delivery in SSA, yet important for any successful PPE implementation. Barriers and facilitators of PPE largely differ from one health system level to another.

**Conclusion:**

Policymakers need to consider the individual and community level contextual factors that influence PPE for effective implementation. Adopting context-specific approaches at all health system levels rather than a one-size-fit-all approach is recommended.

## Highlights

• Public-patient engagement is crucial for health and health systems improvement but lacking evidence synthesis in Sub-Saharan Africa.
• Enhancing engagement requires identifying key barriers and facilitators to PPE implementation.
• Barriers and facilitators of PPE in Sub-Saharan Africa largely differ from one health system level to another.
• Adopting context-specific approaches at all health system levels rather than a one-size-fit-all approach is critical for engagement in the sub-Saharan African context.
• Policymakers need to consider individual and community contextual factors that influence PPE for effective implementation.

## 1 Introduction

Patient-Public Engagement (PPE) is considered central to implementing any community or public health intervention as it promotes mutual sharing of information, resources and ideas between users of health services and health professionals [1]. The Alma-Ata Declaration, as reinforced in the Ottawa Charter and Jakarta Declarations strongly reaffirm governments’ commitment globally towards PPE as a tool to drive health system improvement [2]. Studies have found PPE enables health care services to become more responsive to the needs of relevant populations [2], [3]. For instance, PPE is found to improve access to quality health care [4], [5]; doctor-patient relationships [6], [7]; accountability [8], [9]; and helping ensure openness, rights and responsibilities in relation to patients, their families, communities, health professionals and the entire health system [2], [10]. Despite evidence of demonstrated advantages of PPE in varying health systems, Sub-Saharan Africa continues to report low levels of engagement and low improvements in health outcome [11]. Previous studies have noted some factors accounting for low levels of identified engagement, as well as facilitators that could drive PPE in Sub-Saharan Africa [3], [12], [13]. In Sub-Saharan Africa, this becomes more challenging with the different and dissimilar socio-economic and politico-administrative characteristics of health systems’ architecture. This emphasises the unique and diverse nature of health systems in Sub-Saharan Africa implying also that the breadth, depth and level of engagement are likely to occur at different levels of the health system with varying facilitators and barriers for health and health systems improvement. Yet, literature has not been synthesised to provide an overview of how the facilitators and barriers could improve health and the health care system. Therefore, the aim of this paper is to synthesise the key barriers and facilitators of PPE within Sub-Saharan Africa as identified from a scoping review of literature to understand its effect on health system improvement.

## 2 Materials and methods

This paper is part of a broader study being conducted to identify PPE strategies, barriers and facilitators and mapping these onto an engagement continuum to understand its larger effect on health system improvement in Sub-Saharan Africa. A comprehensive scoping review protocol on this review detailing the methodology has been published [14], and a separate component describing the nature of PPE strategies for health systems improvement has been published. Briefly, the review was guided by Arksey and O’Malley’s guidelines for conducting scoping reviews [15], [16], [17]. Results have been presented using the Preferred Reporting Items for Systematic Reviews and Meta-Analysis extension for scoping reviews (PRISMA-ScR) [18].

The scoping review questions for this paper, informed by the Population, Concept and Context (PCC) framework [18] are:

1. “What are the key drivers of, and barriers to, implementing PPE in Sub-Saharan Africa;
2. What are the current knowledge gaps about PPE in Sub-Saharan Africa?” [14] (p.4)

## 3 Data analysis

We analysed the data using thematic framework analysis [19], [20], [21]. All identified barriers and facilitators were further categorised according to the health system level as previously applied in a review conducted in low and middle-income countries (LMICs) [22]. The mapping of barriers and facilitators onto different health system levels was undertaken by the lead author and independently verified by two members of the research team.

## 4 Results

**Fig. 1:**
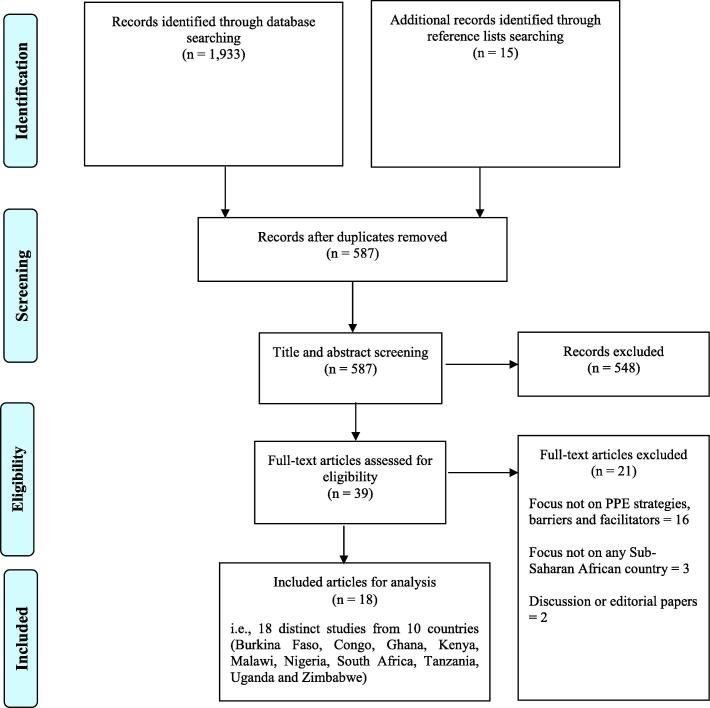
PRISMA flow chart summarising literature search and selection of articles.

In total, 1948 articles were identified in the scoping review; 1933 through database searching using the search criteria mentioned previously, and 15 through hand-searching reference lists of identified articles (Fig. 1). Following removal of duplicate records, the number of articles reduced to 587. A further 548 papers were excluded following the title/abstract screening, leaving 39 papers for full text screening. After applying the inclusion and exclusion criteria, 21 articles were excluded (16 were not focused on PPE strategies, barriers or facilitators; 3 not focused on sub-Saharan Africa; and 2 were discussion or editorial papers). Therefore, 18 articles were retained for final synthesis.

## 5 Characteristics of the included studies

The 18 eligible papers reported on 18 studies, identified from ten Sub-Saharan African countries (Table 1). The majority of studies (n = 14) used qualitative methods [3], [8], [13], [23], [24], [25], [26], [27], [28], [29], [30], [31], [32], [33], one was quantitative [34], and three were mixed methods [12], [35], [36].

**Table 1:** Summary of publications on PPE strategies in Health System Improvement in SSA.

**Author (s) and Year of publication**	**Country of origin**	**Aims/Purpose**	**Study Design and Sample**	**Intervention setting**	**Health System Level**
Adongo et al. 2013 [23]	Ghana	To assess the impact of male involvement in Family Planning in Northern Ghana	Qualitative descriptive design 90 participants via 12 focus group discussions 59 in-depth interviews	Maternal Health Services (Family Planning)	Individual Community Macro/Strategic
Angwenyi et al. 2014 [35]	Kenya	To share community engagement experiences in an on-ongoing paediatric malaria vaccine trial conducted in three study sites of Kilifi County, Kenya	Mixed method descriptive design 25 participants via focus groups 32 participants via in-depth interviews 200 through observational surveys	Malaria Vaccine Trial	Individual Community Macro/Strategic
Baatiema et al., 2013 [3]	Ghana	To explore the PPE process of a community-based health planning and services programme in the Upper West Region of Ghana and evaluate the perspectives of the local stakeholders on their participation in the programme and its impact on health care delivery	Qualitative study applying Spider-gram theory to measure extent of participation 17 participants via in-depth interviews 17 participants involved in 2 focus group interviews	Primary Health Care	Individual Community Macro/Strategic
Campbell et al., 2008 [13]	South Africa	To report on the perceptions of the community on a 3-year programme that seeks to promote grassroots’ responses to HIV/AIDS – mainly through rural health volunteers in KwaZulu-Natal, South Africa	Qualitative study involving 12 participants via in-depth interviews and 34 participants involved in 5 focus group discussions	HIV/AIDS Prevention	Individual Community Macro/Strategic

**Author (s) and Year of publication**	**Country of origin**	**Aims/Purpose**	**Study Design and Sample**	**Intervention setting**	**Health System Level**

Dougherty et al., 2018 [36]	Ghana	To examine how a Community Benefit Health (CBH) programme influenced the outcomes of maternal health services through continuous sustenance of community-level support among the social networks of women	Mixed methods study with 1746 participants involved in questionnaire survey and 183 participants via in-depth interviews & focus group discussions	Maternal Health Services	Individual Community Macro/Strategic
Kamanda et al. 2013 [25]	Kenya	To describe the approaches and principles of Community-Based Participatory Research through harnessing grassroots power in conducting public health research in Sub-Saharan Africa	Randomised controlled trial with semi-annual assessment of 3130 participants	Community Public Health Research	Individual Community Macro/Strategic
Mafuta et al., 2015 [26]	Congo	To explore how health care providers respond to concerns of women through an existing social accountability mechanism in a local setting	Exploratory study involving two health zones with 48 participants interviewed	Maternal Health Services	Individual Community Macro/Strategic
Musesengwa and Chimbari, 2017 [27]	South Africa & Zimbabwe	To document the experiences of members in Community Engagement processes during a project implementation in two countries	Qualitative case study approach involving 102 participants via focus groups and 66 participants via interviews	Malaria and Bilharzia	Individual Community Macro/Strategic
Riehman et al., 2013 [32]	Kenya	To examine the impact of Community-Based Organisations on community and individual level health outcomes, focusing on perceptions, awareness, knowledge, sexual risk behaviours of HIV/AIDS	Quasi-experimental cluster design with multi-method data collection involving 4378 adult respondents	HIV/AIDS prevention	Individual Community Macro/Strategic

**Author (s) and Year of publication**	**Country of origin**	**Aims/Purpose**	**Study Design and Sample**	**Intervention setting**	**Health System Level**

Tancred et al., 2017 [12]	Tanzania	To examine the complexity of community-level quality improvement in health by building capacities of community members to use quality improvement behavioural change towards enhancing maternal and newborn health in Tanzania	Mixed methods involving 83 participants interviewed or involved in focus groups, and quantitative data from secondary sources	Maternal and Newborn Health Services	Individual Community Macro/Strategic
Tindana et al., 2011 [33]	Ghana	To describe the community engagement practices frequently used during implementation of health projects or research through the Navrongo Health Research Centre of Ghana, and to identify the underlying cultural norms that informed those community entry practices	Qualitative case study design involving 116 participants in focus groups and 20 involved in in-depth interview	Public Health Research	Individual Community Macro/Strategic
Meiring et al., 2019 [31]	Malawi	To describe community and stakeholder engagement practices prior and during the typhoid conjugate vaccine trial, drawing lessons from the challenges and its impact on the health outcomes	Qualitative research design within a randomised controlled trial with 380 participants involved in focus groups, interviews, other engagement meetings	Typhoid Vaccine Trial	Individual Community Macro/Strategic
Yeboah and Jagri, 2016 [8]	Ghana	To identify the factors that constrain or facilitate community engagement activities during the implementation of the community-based Health Planning and Services (CHPS) programme in the central region of Ghana.	Qualitative case study design involving 103 participants via questionnaire survey, 8 participants via interviews, 1participant via informal discussion	Primary Health Services/Maternal Health	Individual Community Macro/Strategic

**Author (s) and Year of publication**	**Country of origin**	**Aims/Purpose**	**Study Design and Sample**	**Intervention setting**	**Health System Level**

Person et al., 2016 [29]	Tanzania	To describe, using human-centred design in community co-designed process to prevent and control schistosomiasis. It also aimed to explore how local knowledge, creativity and experiences could be used to design community-owned structural and behavioural interventions to reduce the spread of Schistosomiasis	Cross sectional study involving 5 focus group discussions with community figureheads, 35 school-based discussions with children, 25 interviews with teachers and 16 parents	Schistosomiasis	Individual
Gregson et al., 2013 [24]	Zimbabwe	To investigate if PPE or community grassroots participation resulted in increasing HIV Testing and Counselling services in Zimbabwe	Prospective cohort study involving 5260 participants interviewed in 2 consecutive rounds of cohort survey	HIV/AIDS Prevention	Individual
Ntshanga et al., 2010 [28]	South Africa	To strengthen community mobilization, awareness, education and involvement to improve TB control by building community-health sector partnership through the establishment of Community Advisory Board	Cross sectional study with a total of 140 participants involved in 2 consultative workshops	Tuberculosis Control and Research	Community
Chilaka, 2005 [34]	Burkina Faso, Ghana, Nigeria, Tanzania & Uganda	To use quantitative values to measure and compare levels of community participation in the Roll Back Malaria programme in five Sub-Saharan African countries	Quantitative cross-sectional analysis of database using Spider-gram theory to assess 503 reported malaria cases across the studied countries (excluding Burkina Faso)	Malaria Control	Community
Sakeah et al, 2014 [30]	Ghana	To examine the role played by community leaders and residents during the implementation of skilled delivery programme and its effect on improving maternal health care in a Ghanaian community.	Intrinsic case study design with a qualitative methodology involving 29 health professional and community stakeholders interviewed	Maternal Health Services	Macro/Strategic

CHPS = Community-Based Health Planning and Services

RBM = Roll Back Malaria

CABs = Community Advisory Boards

CHV = Community Health Volunteers

CHW = Community Health Workers

HIV/AIDS = Human Immunodeficiency Virus/Acquired Immunodeficiency Syndrome

## 6 Main findings

This review adapted Charles and DeMaio’s [37] multidimensional framework of engagement in health care decisions, as previously applied by George et al. [22] to categorise the identified barriers and facilitators of PPE into Individual-level factors, Community-level factors, and Macro/Strategic-level factors. The Individual level factors are those that directly relate to an individual, including personal factors, which facilitate or impede pathways to successful PPE. Community-level factors are those that relate to a community rather than just an individual. Lastly, the Macro/Strategic-level factors are those relating to strategic governmental policies or larger politically related factors that influence PPE.

Thirteen papers reported on facilitators and barriers of PPE at all the three health system levels (individual, community and macro/strategic) [3], [8], [12], [13], [23], [25], [26], [27], [31], [32], [33], [35], [36](Table 1). The remaining five papers reported on barriers and facilitators at either the individual [24], [29], community [28], [34] or Macro/strategic levels only [30].

## 7 Individual-level facilitators and barriers

At the individual level, four articles reported that intrinsic motivation or people’s willingness to participate is a major PPE facilitator in Sub-Saharan Africa [3], [12], [23], [25]. As noted in previous studies, PPE or community-based health intervention programmes largely depend on people’s willingness to support the programme [12]. For instance, in a study conducted in Ghana, it was reported that individual willingness was key to the success of a PPE programme that aimed at involving males in family planning services [23]. Therefore, prior to commencement of PPE, there is the need to intensify education and sensitisation to boost people’s interest and willingness to participate in the programme [3].

Another individual-level facilitator identified was the opportunity PPE offers people to grow their personal and professional competencies as well as also gaining recognition and respect from their community [12], [13], [26], [28], [33]. In a study conducted in Tanzania, Tancred et.al noted that people mostly view PPE as an opportunity to develop their knowledge and professional understanding for the health care system [12]. Other studies from Kenya and Ghana also reported that people living in rural communities in Sub-Saharan Africa see their involvement in PPE activities as an honour that comes with respect and recognition from their family and community [8], [25], [33], [36].

Again, people’s interest to develop their leadership skills and knowledge through their representation on Community Advisory Boards (CABs) is a major facilitator for PPE implementation across Sub-Saharan Africa [27], [31], [32], [35]. For instance, in Malawi, it was observed that one key facilitator for increasing voluntary participation in CABs was exploring individual’s personal interest in developing their leadership skills and knowledge. It was explained that CAB membership provided the opportunity to be recognised and selected for future community-based programmes or initiatives [31].

In addition, people’s sense of ownership for community-based health programmes was noted as an important facilitator for PPE in Sub-Saharan Africa [27], [30], [33], [34]. This is particularly common in rural communities where sense of communalism is often strong. Individuals in these communities mostly take keen interest in programmes aimed at promoting health and wellbeing of their community [3], [26], [33].

In relation to individual level barriers, it was reported that lack of appropriate training, education and skill was a major setback for people’s participation in PPE [27], [31], [32], [35]. Our review identified most community members chose not to be part of a PPE programme until they were offered the requisite training. For instance, in a study conducted in Kenya and South Africa, it was revealed that opinion leaders selected to lead PPE programmes were initially reluctant to be part of the programme until they were assured of training support [27], [35].

Other studies also identified different individual-level barriers such as inadequate information or lack of clarity on the roles and responsibilities of community representatives [8], [12], [28], and lack of logistical and financial support for the work of Community Health Volunteers (CHVs) [26], [28], [35]. Particularly, this review notes that CHVs play an important role in PPE with their focus on improving health and the health delivery system in most rural communities in Sub-Saharan Africa [3], [13], [26]. However, logistical and financial constraints can be a key barrier to their work. Although the focus of CHVs is not to receive financial incentives, it is equally important to adequately resource their roles, logistically and financially to facilitate their voluntary work within the community [28] Table 2.

**Table 2:** Summary of Individual-Level Facilitators and Barriers to PPE.

	**Individual-Level Factors**	
	**Facilitators**	**Barriers**
1.	Intrinsic motivation – willingness to participate	Lack of appropriate skills, training and education
2.	Personal and professional growth, recognition and respect	Insufficient information regarding roles and responsibilities
3.	Development of leadership skills and knowledge	Lack of logistical and/or financial support for work, particularly for CHVs
4	Sense of ownership for PPE initiatives	

## 8 Community-level facilitators and barriers

At the community level, one key PPE facilitator largely reported in the included studies was aligning PPE to suit the cultural norms of the people as well as designing the programme to fit the local needs of the community [3], [13], [25], [26], [28], [33], [35]. Most studies reviewed in this paper reported that any PPE designed and implemented to suit the cultural norms of the community is likely to be accepted and supported [25], [33].

Additionally, the use of pre-existing community structures such as the indigenous/traditional health care systems (e.g herbal healers, spiritualists and faith healers) were identified as another important community-level facilitator for PPE in Sub-Saharan Africa. Evidence of this was reported in seven articles, noting that using such pre-existing community structures promote trust as well as gaining grassroot support for PPE programme [3], [13], [25], [26], [28], [33], [35]. For instance, it was noted in a study conducted in Congo that integrating Traditional Birth Attendants (TBAs) into a new maternal health programme was key to avoiding conflicts between the TBAs and health authorities. This also resulted in improved maternal health outcomes, particularly, in the two studied communities as maternal health cases were referred early from the TBAs to the health facilities [26].

Again, the expected benefits of a health intervention to the community has been widely reported as a key driver for PPE in Sub-Saharan Africa [8], [31], [34]. An example was cited in a study conducted in Central Region of Ghana, where perceived benefits of constructing a clinic was a major facilitator for an increased community interest and support for the programme. Specifically, the community mobilised additional resources for the clinic construction because it expected the clinic to help address their major difficulties in accessing health care [8]. Therefore, in situations where PPE encounter community resistance, it is important to highlight extensively the expected benefits of the programme to the community [34].

It was again noted in this review that the democratic and receptive position of trained health workers towards the views and opinions of non-health workers was a major facilitator for PPE [22]. Five articles reported that positive attitudes of health professionals towards community views contributed significantly to shaping community interest and involvement to improve health and the health care system [8], [13], [25], [31], [32]. In a study from Ghana, it was found that partnership between health professionals and a community to construct a clinic through the Community-based Health Planning and Services (CHPS) programme realised maximum support from the community, including mobilising funds for the construction of the clinic. However, failure of the health care system to sustain this shared partnership with the community during ongoing functioning of the clinic affected the ultimate goal of the programme [8].

Community-level barriers to effective PPE include a lack of trust or support for community representatives [3], [27], [28]. This was noted as particularly common in situations where communities were not involved in the selection of representatives to CABs [28]. In many instances, it was found that CAB members were selected, in isolation from the community, by traditional authorities, programme managers/health care authorities or politicians [27], [28].

A lack of trust between health professionals and the wider community was also noted as a major barrier to PPE implementation in Sub-Saharan Africa [8], [12], [28], [33]. Several reasons have been attributed to the mistrust. These include, health professionals ignoring community inputs, suggestions and concerns [26], [35]; lack of accountability [8], [26]; top-down approach to decision making [3], [12], [31]; and lack of confidence in the abilities of the community [32], [33].

For example, a study conducted in Congo noted that ignoring community suggestions and concerns at the early stages of a PPE programme potentially impeded the early success of the programme as this conflicted with the cultural and local needs of the community [26] Table 3.

**Table 3:** Summary of Community-Level Facilitators and Barriers to PPE.

	**Community-Level Factors**	
	**Facilitators**	**Barriers**
1	Similarities with cultural norms or fit with local needs	Lack of trust or support for community representatives or CAB
2	The use of pre-existing community structures	Belittling of community inputs or suggestions
3	Expected positive benefits of the health intervention to the community	Lack of confidence by health professional/programme implementers in the abilities of communities
4	Positive attitudes of health workers or programme implementers towards shaping community interest	

## 9 Macro/strategic-level facilitators

At the macro/strategic level, democratic political inclinations of national governments, as well as open and transparent communication across all levels were noted as key facilitators of PPE. In a study conducted in Tanzania, national government’s ideological views towards shared decision-making was considered a key facilitator for PPE in Sub-Saharan Africa [12]. Although, this has not been widely explored in most Sub-Saharan African countries, this review found ideological inclinations of some governments in the sub-region actively promoted decentralised decision making which allows communities to be part of core health care decision making at the local or primary health level [12], [36].

Another reported PPE facilitator at the macro/strategic level included having effective policy framework and strategy for open and transparent communication across all key stakeholders. This helps prevent communication gaps that could promote active community involvement particularly during implementation of community-based health interventions [30].

A lack of effective accountability policy was also identified as a major reason for mistrust and tension between health professionals and communities. This was summarised in an opinion expressed by community leaders during a study conducted in the Central Region of Ghana where the community demanded financial accountability of the facility they have actively participated in constructing before they can further support maintaining the facility [8] Table 4.

**Table 4:** Summary of Macro/Strategic-Level Factors.

	**Macro/Strategic-Level Factors**	
1.	Transparency and open communication	Dominance of top-down approaches to decision making and professional dominance of health workers
2.	Changes in political inclinations or ideologies towards allowing decentralised policy and decision making in the health sector	Lack of open accountability to community

## 10 Discussion

The synthesis presented in this paper is part of a broader scoping review, which comprehensively examines various PPE strategies in Sub-Saharan Africa to understand its broader effect on health system improvement. The significant contribution of this paper is its ability to identify the various barriers and facilitators of PPE at each level of the health system. The review also takes into consideration, the unique and diverse nature of the various health system levels, as well as the varying effects the identified barriers and facilitators have on PPE implementation in Sub-Saharan Africa. We identified no other reviews that have synthesised evidence representing such broad range of PPE barriers and facilitators and mapped according to the three health system levels (individual, community and macro/strategic).

At the individual level, intrinsic motivation or people’s willingness to participate was often identified PPE facilitator. This was particularly observed in rural communities where sense of communal support for community-based programmes was found to be strong [3], [23], [27]. However, it was noted that certain factors may act as barriers to intrinsic motivation. For instance, in a study conducted in Kenya, community members elected to a CAB were motivated to be part of the PPE programme, but a lack of appropriate training and sensitisation adversely affected their initial interest [25]. Similar observations were also made in another study conducted in South Africa [28]. Our review, therefore, suggests a comprehensive training and sensitisation prior to PPE implementation to boost people’s knowledge and confidence to willingly get involved, particularly in urban communities where sense of communal support is considered low [1], [25], [28], [38].

At the community level, designing PPE to fit local needs and cultural norms of the community was frequently reported as a key facilitator [3], [13], [25], [26], [28], [33], [35]. Other factors, such as using pre-existing community structures [3], [13], [25], [26], [28], [33], [35] and positive attitudes of health workers, were also reported community-level facilitators of PPE [8], [13], [25], [31], [32]. Similar findings were made in other studies conducted in low-to-middle income countries [22], [39], [40], [41].

Regarding barriers to PPE implementation in Sub-Saharan Africa, we identified lack of trust and support for externally-appointed community representatives on CABs [3], [27], [28]; insufficient information on CAB roles [28]; lack of accountability [8], [30]; inappropriate training for community representatives on CABs [27], [31], [32], [35]; and an emphasis on top-down decision making [3], [12], [31]. Most reported barriers were associated with CABs. As a result, we suggest that selection of CAB members must be an open, transparent and all-inclusive process that ensures social capital between community representatives on PPE advisory boards and the community at large [3]. In addition, we recommend the selection of community representatives must be based on traditional kinship structures as well as working through community-based organisations to ensure no key group is excluded in the engagement process [33]. We further recommend regular community sensitisation and education particularly on the role of the CABs.

We again identified that policies regulating most public health care facilities across all levels of the health system in Sub-Saharan Africa largely excludes involvement of beneficiary communities from financial and managerial accountability [8]. For instance, in the study conducted in the Central Region of Ghana, it was found that although the clinic was constructed with the assistance of the community, the health workers managing the clinic, by policy, were accountable to the District Health Directorate and not to the community health management team. This may have increased the community’s apathy towards further supporting the facility as they felt ignored in the accountability process [8], [42].

Lastly, we also noted most community-based health PPE interventions in Sub-Saharan Africa struggled to achieve their goals when health programmes were hierarchically imposed on communities [12]. Although PPE is not strictly a bottom-up approach, collaborating with communities to design systems appear crucial [3], [30], [37], [43].

## 11 Recommendation for improvement in practice

Barriers and facilitators of PPE largely differ from one health system level to another. Although these may be closely linked, we noted that the one-size-fit-all approach to addressing PPE issues across all levels of the health system may not be effective. Therefore, we recommend the need for policy makers to consider the individual and community contextual factors that influence PPE for effective implementation.

Further, we recommend the inclusion of community health committees in the accountability processes, particularly in facilities where community engagement was key in its establishment.

Finally, we advocate for policy-making processes that ensure shared decision-making responsibility between the community and health care professionals in relation to implementation of community-based health interventions rather than a reliance on top-down decision-making.

## 12 Strengths and limitations

Our review focused on only English language peer-reviewed articles, which may have excluded PPE studies reported in the grey literature and other non-English language journals. Particularly, we note that there may be PPE initiatives in Sub-Saharan Africa that are never published in scientific peer-reviewed journals. However, we sourced and reviewed a large number of potentially eligible papers on PPE in Sub-Saharan Africa. In addition, the framework adapted to categorise the barriers and facilitators, which was independently verified, ensured the eligible papers were analysed within the context of the health system levels to understand its overall effect on health system improvement.

Papers eligible for this review did not necessarily report on all important barriers or facilitators of PPE. For instance, apart from two studies [3], [26] that discussed the effect of demographic characteristics on PPE, none of the selected papers focused on this aspect. However, literature has widely reported of the importance of these demographic characteristics to the Sub-Saharan African socio-cultural traditions [7], [44], [45]. Hence, further research seems warranted to investigate the specific impacts of these demographic characteristics, and other unreported barriers and facilitators of PPE in Sub-Saharan Africa. Finally, we note the unique and diverse nature of health systems of each Sub-Saharan African country, hence, the possibility of not being able to adequately apply findings to every country in Sub-Saharan Africa. However, our study identified literature from 10 Sub-Saharan African countries, which means our findings are likely to be applicable to many health systems in the region, particularly those sharing similar health system capacity needs and challenges to these countries.

## 13 Conclusion

Whilst few reviews have been conducted on PPE in Sub-Saharan Africa, these have not previously focused on barriers and facilitators of PPE. It is, however, noted that for any successful PPE implementation, identifying the key barriers and facilitators is important. Our review, has, thus identified these barriers and facilitators and also analysed its effect on improving the health delivery system in Sub-Saharan Africa. The identified barriers and facilitators were categorised according to a framework of health system levels (individual, community and macro/strategic) which also enabled us to analyse them according to their unique and diverse nature, as well as their varying effect on PPE implementations in Sub-Saharan Africa.

Intrinsic motivation was found as the most reported individual level facilitator, although, we identified a lack of training and sensitisation could become a major barrier to this. Hence, our recommendation for extensive training and sensitisation prior to implementing PPE. We again noted that most reported PPE barriers, particularly at the community level were found to be associated with CABs; with majority of the reported incidences related to external appointment of community representatives to health advisory boards. We therefore suggested an open, transparent and all-inclusive selection process for community representatives on PPE advisory boards.

Also, we noted that policies regulating most public health care facilities across all levels of the health system in Sub-Saharan Africa largely excludes involvement of beneficiary communities from financial and managerial accountability. This potentially increases community apathy, particularly towards sustaining community-based health interventions. Thus, we recommend a policy change, particularly at the community level to include community health management committees in the accountability processes, especially in facilities where community engagement was key in its establishment.

We also found that most health care decisions or policy directives from the macro/strategic level to the community level take a ‘top-down’ approach. This potentially affects the opportunities for beneficiary communities to adequately participate in health care decisions and ensure service delivery accords with community needs and preferences. Although, we admit PPE is not necessarily a ‘bottom-up’ approach, there should be conscious effort made to prioritise patients or community preferences when making health care decisions.

Lastly, we noted the barriers and facilitators of PPE largely differ from one health system level to another. Hence, we acknowledge the differences in the health systems of Sub-Saharan African countries, and note that the ‘one-size-fits-all’ approach to addressing PPE issues across all levels of the health system may not be effective. However, it is important to think about barriers and facilitators to PPE initiatives at the different health system levels in specific country contexts prior to implementation. Therefore, we recommend policy makers carefully consider the individual and community contextual factors that influence PPE to support effective implementation and improved health outcomes in Sub-Saharan Africa.

### CRediT authorship contribution statement

**Samuel Egyakwa Ankomah:** Conceptualization, Methodology, Writing – original draft, Visualization, Investigation. **Adam Fusheini:** Validation, Writing – review & editing. **Sarah Derrett:** Data curation, Writing – review & editing.

## Declaration of Competing Interest

The authors declare that they have no known competing financial interests or personal relationships that could have appeared to influence the work reported in this paper.
